# Comparison of high-intensity laser therapy and combination of transcutaneous nerve stimulation and ultrasound treatment in patients with chronic lumbar radiculopathy: A randomized single-blind study

**DOI:** 10.12669/pjms.343.14345

**Published:** 2018

**Authors:** Emine Kolu, Raikan Buyukavci, Semra Akturk, Fatma Eren, Yuksel Ersoy

**Affiliations:** 1 Emine Kolu, Inonu University, Faculty of Medicine, Department of Physical Medicine and Rehabilitation, Malatya, Turkey; 2 Raikan Buyukavci, Inonu University, Faculty of Medicine, Department of Physical Medicine and Rehabilitation, Malatya, Turkey; 3 Semra Akturk, Inonu University, Faculty of Medicine, Department of Physical Medicine and Rehabilitation, Malatya, Turkey; 4 Fatma Eren, Inonu University, Faculty of Medicine, Department of Physical Medicine and Rehabilitation, Malatya, Turkey; 5 Yuksel Ersoy, Inonu University, Faculty of Medicine, Department of Physical Medicine and Rehabilitation, Malatya, Turkey

**Keywords:** High intensity laser therapy, Lumbar radiculopathy, transcutaneous nerve stimulation, Ultrasound

## Abstract

**Objective:**

To compare the effects of high-intensity laser therapy (HILT) and a combination of transcutaneous nerve stimulation (TENS) with ultrasound (US) therapy on pain and functionality in patients with chronic lumbar radiculopathy.

**Methods:**

This prospective randomized comparative study was conducted in Department of physical medicine and rehabilitation, Turgut Ozal Medicine Center, Malatya, Turkey from April 2016 to September 2016. A total of 54 patients with chronic lumbar radiculopathy were enrolled in this study. The patients were randomly divided into two groups: Group 1 (n:27) received 10 sessions of a combination of hot pack, TENS, US and exercise, and Group 2 (n:27) received hot pack, HILT and exercise. The outcomes measured were low back with unilateral leg pain level measured by visual analog scale (VAS) and functionality measured with the Oswestry Disability Index (ODI) at the end of the therapy and four weeks later. p-value less than 0.05 considered statistically significant.

**Results:**

In two groups, VAS (low back with unilateral leg pain) and ODI scores showed significant changes. At the end of the 2 weeks intervention, participants in Group-1 showed a significantly greater decrease in pain than participants in Group-2. Statistically significant differences in pain variation and functionality (VAS and ODI) were observed four weeks after treatment sessions for participants in the TENS+US therapy group compared with participants in the HILT group.

**Conclusion:**

HILT and TENS+US combined with exercise were effective treatment modalities in decreasing the VAS and ODI scores. TENS+US combined with exercises were more effective than HILT combined with exercise.

## INTRODUCTION

Low back pain is one of the most common musculo-skeletal system pains that cause loss of work power and negatively affects quality of life. The prevalence of low back pain, observed in every culture and ethnic group, is reported as nearly 84%.^1^ The rate of chronic back pain is about 10%.^2^

Discogenic low back pain, whether accompanied by radicular symptoms or not, is one of the common causes of low back pain.^3^ There are a variety of conservative treatment methods for low back pain, led by lumbar radicular symptoms. There is a wide spectrum of conservative treatment options including patient education, behavioral therapies, back school, exercise, and physical therapy modalities such as traction, superficial heaters, deep heaters (short wave diathermy, ultrasound etc.), transcutaneous electrical nerve stimulation (TENS), and laser.^4^

Laser is a pain-free and non-invasive treatment modality.^5^ It is used in many acute and chronic painful conditions. HILT therapy is a type of Nd YAG laser with 1064 nm wavelength. Light with slow chromofors and low level is absorbed and deep tissues are affected without radiation. High intensity lasers may affect deeper tissue as they have shorter emission time and longer emission intervals compared to low intensity lasers.^6^ In recent times, the use of high intensity laser for physical therapy has been shown to significantly reduce pain with a variety of causes.^7^,^8^ The anti-edematous, anti-inflammatory and analgesic effect of Nd YAG laser for patients with pain has been determined by studies.^9^ Though there is no universal consensus to clearly explain the effect mechanism of laser, it is accepted as having three effects; photothermal, photochemical and photomechanic.^10^,^11^

In the literature there is no consensus on the application duration, pulse power, energy dose and frequency to be used for laser treatment of patients. There are a few studies to date on the effects of HILT therapy on cervical radiculopathy, frozen shoulder, lateral epicondylitis, carpal tunnel syndrome, myofascial pain syndrome, low back pain, gonarthrosis, post-mastectomy and lumbar discopathy pain.^7^,^8^,^12^,^13^

There are studies in the literature showing the efficacy of physical therapy modalities and HILT in patients with chronic low back pain.^14^-^16^ However, in patients with chronic lomber radiculopathy, the literature is limited in terms of HILT treatment.

In this study we aimed to compare TENS and Ultrasound combination, commonly used in routine practice, with the new non-invasive treatment method of HILT in terms of effects on pain and functionality for patients with chronic lumbar radiculopathy. At the same time, we aimed to suggest an appropriate and effective treatment proposal for patients with chronic lomber radiculopathy for HILT, which is one of the new treatment options.

## METHODS

This prospective single-blind randomized study included patients, visiting the Department of Physical Medicine and Rehabilitation, Turgut Ozal Medicine Center, Malatya, Turkey from three 3 months low back with unilateral leg pain and clinical signs of radicular lesion in dermatomal distribution and/or myotomal muscle weakness and/or diminished reflexes in lower limbs. Lumbar spinal root pressure was detected by MRI.

The local hospital ethics committee approved the study. Patients agreeing to participate in the study provided an informed consent form. Exclusion criteria for the study were previous history of spinal surgery, sequestrated disk hernia on MRI, steroid injection and/or physical therapy for the lumbar region within the last four weeks, inflammatory rheumatic disease, cardiac pacemaker, continuing or previous malignancy history and pregnancy.

Patients included in the study were randomly divided into two groups. The patients in Group 1 had a total of 10 sessions of hot pack, TENS and ultrasound treatment applied over two weeks for five days each week. Patients had hot pack applied to the lumbar paravertebral area for 20 minutes, along with TENS application in conventional mode for 20 minutes at 70 Hz frequency and 100 microsecond wave length. Later again in the lumbar paravertebral region, patients had therapeutic ultrasound treatment of 1.3 watt/cm2 power, 1 mHz frequency applied with a US device (BTL-4825S Kombi Topline) for 10 minutes continuously.

The second group had high intensity laser device (BTL 6000) used after 20 minutes hot pack application for five days per week over two weeks for a total of 10 sessions of high intensity laser treatment. The device was set to 25 Hz frequency, 10 watt power with 12 j/cm2 dosage to the lumbar region over 25 cm2 area for four minutes biostimulation mode, followed by continuous mode for 6 minutes with 7 watt power and 120 j/cm2 dosage.

In addition, an isometric lumbar exercise program was initiated by the same physiotherapist to be performed with five repetitions in each set (modified straightening and pelvic tilt exercises) in Groups-1 and 2 during the therapy duration. The repetitions of both sets were increased up to ten, provided that this did not increase the patient’s pain.

### Assessment of pain

The patients were assessed with the visual analog scale (VAS) for low back with unilateral leg pain at rest, when moving and at night. Accordingly on a 10 cm line, the 0 point was accepted as no pain while the 10 point was accepted as maximum pain. Patients were asked to indicate the severity of low back pain on this line. Later the distance between the 0 point and the marked point was measured with a ruler.

### Oswestry Disability Index

This comprises 10 questions assessing pain, personal care, lifting, walking, sitting, standing, sleeping, social life, travel and degree of pain variation, with each scored from 0 to 5. Maximum points are 50 and total score is multiplied by two to provide a percentage result. The evaluation is made with the formula: point/total score (50) x 100 = %. This form used to assess treatment results and compare different treatments in chronic low back pain patients and Turkish validity and reliability has been proven.^17^

**Fig.1:**
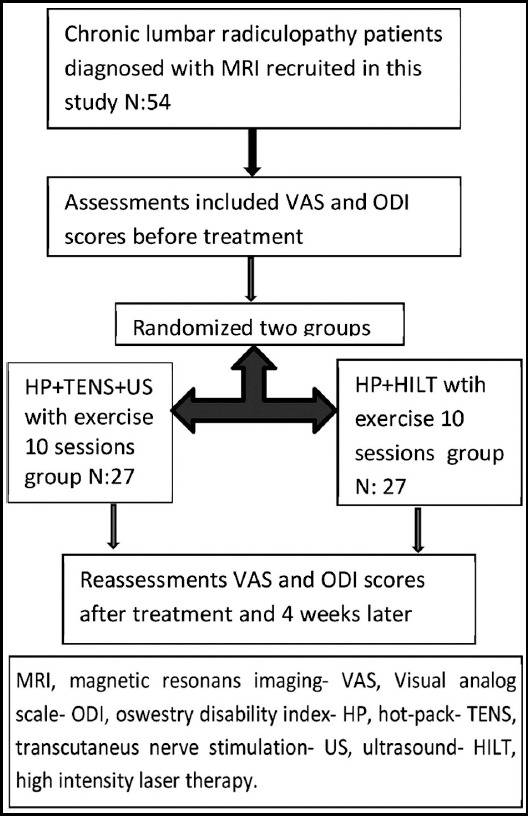
Flow diagram of the study.

The pateints were assessed by the same blinded doctor before treatment, after treatment and four weeks later for pain with VAS and for functional state with ODI. Flow diagram of the study has been given Fig.1.

### Statistical Analysis

It was calculated that a total of 54 individuals should be taken, with at least 27 subjects from each group when α = 0.05 and 1-β = 0.80 were taken in the power analysis performed.

For statistical analysis of research data, SPSS for Windows version 17.0 software was used. Data related to quantitative variables are given as arithmetic mean ± standard deviation (sd) and min-max, while data related to qualitative variables are given as number (n) and percentage (%). The one-way ANOVA test was used for comparisons of the parametric continuous data. The Kruskal-Wallis test was used for the nonparametric continuous data. A repeated measures ANOVA was used to analyze the changes in variables. Significant differences were determined by Bonferroni post hoc tests. A value of p<0.05 was accepted as statistically significant.

## RESULTS

A total of 54 patients participated in this study. Group 1 (HP+TENS+US) consisted of 27 patients with mean age 50.14±15.55, while Group 2 (HP+HILT) consisted of 27 patients with mean age 53.40±10.57 years. The female/male distribution was 20/7 in Group-1 and 16/11 in Group 2, while BMI was 26.9±3.65 kg/m^2^ in Group-1 and 26.7±3.65 kg/m^2^ in Group 2. There was no difference between the two groups in terms of age and BMI (p>0.05). In terms of pain duration, there was no difference between the two groups (Group-1: 3.66±2.89 years, Group 2: 5.18±5.13 years, p>0.05) (Table-I).

**Table-I:** Patients demographic datas.

	Group 1 (n=27) HP+TENS+US		Group 2 (n=27) HP+HILT		P value

	Mean±SD	Min-Max	Mean±SD	Min-Max	
Age (year)	50.14±12.55	19-64	53.40±10.57	22-65	0.363
BMI (kg/m^2^)	26.9±3.65	17-33	26.7±3.65	21-34	0.878
Duration of illness (age)	3.66±2.89	0.5-20	5.18±5.13	0.5-15	0.530

**BMI:** Body Mass Index

The VAS and ODI scores were similar in the two groups before treatment (p>0.05) (Table-II). The comparison of parameters in Group-1 and Group-2 before treatment and at the end of therapy revealed significant difference changes in VAS and ODI scores (p<0.05).

**Table-II:** Baseline VAS scores and Oswestry Disability Index score for two groups.

Before Treatment	Group 1 HP+TENS+US	Group 2 HP+HILT	P value
Resting VAS score	4.33±1.79	4.29±1.75	0.965
Moving VAS score	8±0.78	7.78±1.06	0.554
Night VAS score	3.25±1.43	3.29±1.26	0.783
ODI score	68.51±14.18	70.22±12.63	0.735

**VAS:** Visuel analog scale, **ODI:** Oswestry Disability Index score.

**P value:** p value obtaining by which comparing statistcally scores of the scales among the groups before treatment.

Assessment of the two groups at the end of treatment and 1 month after treatment found that the moving VAS score and ODI score were statistically significantly lower in Group 1 (HP+TENS+US) (p<0.05) (Table-III).

**Table-III:** Changes in VAS scores and Oswestry Disability Index score in the two groups at the and of the treatment and 1 month later after therapy.

	Group 1			Group 2				

	Pre treatment	Post treatment	1.month	Pre treatment	Post treatment	1.month	P^1^ value	P^2^value
RestingVAS score	4.33±1.79	2.66±1.30	2.70±1.40	4.29±1.75	2.90±1.19	2.85±1.16	0.283	0.486
Moving VAS score	8±0.78	4.33±1.27	4.22±1.05	7.78±1.06	5.18±1.38	5.29±1.51	0.027*	0.011*
Night VAS score	3.25±1.43	2.25±1.16	2.25±1.22	3.29±1.26	2.66±1.17	2.81±1.24	0.198	0.091
ODI score	68.5±14.1	42.5±12.8	45.1±13.0	70.2±12.6	51.4±12.6	54.5±14.6	0.014*	0.014*

***VAS:*** Visuel analog scale, ***ODI:*** Oswestry Disability Index score.

***P^1^:*** p value obtaining by which comparing statistcally scores of the scales among the groups after treatment.

***P^2^:*** p value obtaining by which comparing statistcally scores of the scales among the groups 1 month later after treatment.

* p<0.05 for the Kruskal Wallis test.

## DISCUSSION

In this present study, patients with chronic lumbar radiculopathy in the high intensity laser treatment (HILT) and ultrasound (US) with transcutaneous nerve stimulation (TENS) combination groups were compared in terms of VAS scores and Oswestry Disability Index (ODI) score. In the two groups, VAS and ODI scores showed significant changes. However, patients receiving TENS+US treatment had a greater reduction in VAS scores and ODI score at the end of treatment and 1 month later compared to the HILT group.

In recent years, high intensity laser treatment has been used for a wide range of painful conditions. The efficacy of the pulsed Nd:YAG laser has been proven in the treatment of many musculoskeletal diseases and it is believed to have anti-inflammatory, anti-edema, analgesic, and reparative effects.^18^ The analgesic effect of HILT is based on different mechanisms of action, including its ability to slow the transmission of the pain stimulus and to increase the production of morphine-mimetic substances in the body.^6^ In addition, it may have a direct effect on nerve structures, which could increase the speed of recovery from conduction block or inhibit Aδ- and C-fiber transmission.^19^ The treatment also increases blood flow, vascular permeability, and cell metabolism.^20^

A recent study by Choi et al. randomly divided patients with chronic low back pain into two groups. One group received conservative treatment (HP+TENS+US), while the other group received 10 minutes of HILT with 1378 mJ/ cm^2^ to the L1-S1 region three times per week after conservative treatment for four weeks. The response to treatment was assessed with VAS and ODI scores before treatment and after treatment and they concluded that the addition of HILT treatment to conservative treatment was more effective on pain and function for chronic low back pain patients. However, the low number of patients in the groups and the lack of assessment of long term efficacy of treatment were given as limitations of the study.^21^

A study by Fiore et al. compared the efficacy of high intensity laser treatment and ultrasound for patients with low back pain.^22^ Each group had VAS and ODI scores assessed after 15 sessions of treatment. There was significant amelioration of the VAS and ODI scores in both groups at the end of treatment. When the laser group is compared with the ultrasound group, it appeared the laser group had significant superiority for VAS and ODI scores. Another study by Boyraz et al. assessed the pain and quality of life with lumbar disc herniation patients receiving three different therapeutic methods of HILT, US and medical treatment. All three forms of treatment were effective; however they concluded HILT and US treatment were effective in the long term.^23^

Non-pharmacological methods including a variety of physical therapy agents are the cornerstone of management of chronic low back pain. Therapeutic ultrasound (US) is among the commonly used physical modalities for treating soft tissue injuries. There is some evidence that therapeutic ultrasound has a small effect on improving low-back function in the short term, but this benefit is unlikely to be clinically important. Evidence from comparisons between other treatments and therapeutic ultrasound for chronic low back pain were indeterminate and generally of low quality.^14^ As with many causes of musculoskeletal pain, for low back pain the combination of therapeutic US with TENS has been shown to be more effective than applications alone for pain and disability.^24^ There are very few studies comparing the TENS and Ultrasound combination commonly used in daily practice by physiatrists with HILT as our study does.

The results of this study show that in addition to the TENS and US combination, among physical therapy agents ensuring reduction in pain and disability for chronic low back pain patients, the new modality HILT treatment is effective; however it was concluded that there is a need for long term monitoring and controlled studies.

### Limitations

Small number of patients because of economic reasons, the lack of evaluation of long term results and the lack of a control group.

### Authors’ Contribution

***EK, RB and FE*** conceived, designed and did statistical analysis & editing of manuscript.

***EK and RB*** did data collection and manuscript writing.

***SA and YE*** did review and final approval of manuscript.
